# Supplementary material for: Capecitabine in Combination with Endocrine Therapy as Maintenance Therapy after Bevacizumab Plus Paclitaxel Induction Therapy for Hormone Receptor-Positive, HER2-Negative Metastatic Breast Cancer: KBCSG-TR1214
Source: Cancers (Basel). 2021 Aug 31;13(17):4399. doi: 10.3390/cancers13174399 (PMC8430728; doi:10.3390/cancers13174399)
Supplement: Supplementary file 1 [file cancers-13-04399-s001.zip › cancers-1261498-supplementary.pdf]

Supplementary materials

		No. of patients	EC	PFS (95%CI)	E	PFS (95%CI)	HR (95%CI)	P-value	Interaction test P-value
Age	<60	46	n=22	11.0 (4.8 - 11.8)	n=24	3.7(1.8 - 5.4)	0.60 (0.32 – 1.12)	0.106	0.74
	≥60	44	n=22	11.5 (5.8 - 14.3)	n=22	5.6(3.7 - 7.4)	0.47 (0.25 - 0.89)	0.021	
ECOG Performance status	0	64	n=31	11.4 (8.3 - 13.4)	n=33	3.9 (3.1 - 6.1)	0.59 (0.35 – 1.00)	0.049	0.54
	1-2	26	n=13	9.9 (5.7 - 28.2)	n=13	4.7 (1.6 - 7.1)	0.43 (0.18 - 1.02)	0.055	
Menopausal status	Pre	20	n=10	11.1 (0.6 - 19.5)	n=10	3.4 (0.5 - 9.7)	0.65 (0.24 - 1.75)	0.388	0.60
	Post	70	n=34	11.2 (5.8 - 13.4)	n=36	4.7 (3.7 - 6.1)	0.48 (0.29 - 0.80)	0.005	
Histological grade	Grade 1	15	n=7	17.2 (4.8 - NA)	n=8	7.8 (1.7 - NA)	0.78 (0.24 - 2.56)	0.683	0.75
	Grade 2+3	47	n=24	11.1 (5.5 - 11.6)	n=23	3.8 (1.8 - 5.4)	0.44 (0.24 - 0.81)	0.008	
	Unknown	28	n=13	10.9 (5.7 - 17.0)	n=15	3.9 (1.6 - 7.4)	0.51 (0.22 - 1.15)	0.106	
PgR	Negative	13	n=6	8.5 (2.0 - NA)	n=7	5.7 (1.7 - 7.1)	0.50 (0.14 - 1.76)	0.282	0.58
	Positive	75	n=37	11.0 (8.3 - 11.8)	n=38	3.9 (3.1 - 6.0)	0.58 (0.35 - 0.94)	0.026	
Prior chemotherapy for AMBC	No	79	n=41	11.1 (8.3 - 13.4)	n=38	4.3 (3.6 - 6.0)	0.54 (0.33 - 0.87)	0.011	0.57
	Yes	11	n=3	5.7 (0.6 - NA)	n=8	4.6 (1.4 - 10.4)	0.56 (0.12 - 2.70)	0.470	
No. of prior endocrine therapies for AMBC	<3	65	n=32	11.3 (5.7 - 13.5)	n=33	5.7 (3.7 - 9.7)	0.63 (0.37 - 1.07)	0.086	0.04
	≥3	25	n=12	10.5 (5.7 - 14.3)	n=13	3.7 (1.6 - 3.9)	0.10 (0.03 - 0.34)	<0.001	
Response to induction chemotherapy	CR+PR	69	n=34	11.5 (8.3 - 14.3)	n=35	3.9 (3.6 - 5.7)	0.43 (0.26 - 0.73)	0.002	0.07
	SD	21	n=10	6.9 (0.6 - 11.1)	n=11	4.8 (1.6 - 10.4)	1.07 (0.43 - 2.65)	0.889	
Endocrine therapy	SERM	19	n=8	11.2 (4.1 - NA)	n=11	3.8 (1.6 - 9.7)	0.48 (0.17 - 1.33)	0.156	0.45
	SERD	12	n=6	5.7 (1.8 - NA)	n=6	4.5 (1.8 - NA)	1.06 (0.32 - 3.52)	0.926	
	AI ± LHRH agonist	55	n=27	11.6 (8.3 - 17.2)	n=28	4.3(2.2 - 5.7)	0.50 (0.28 - 0.89)	0.018	

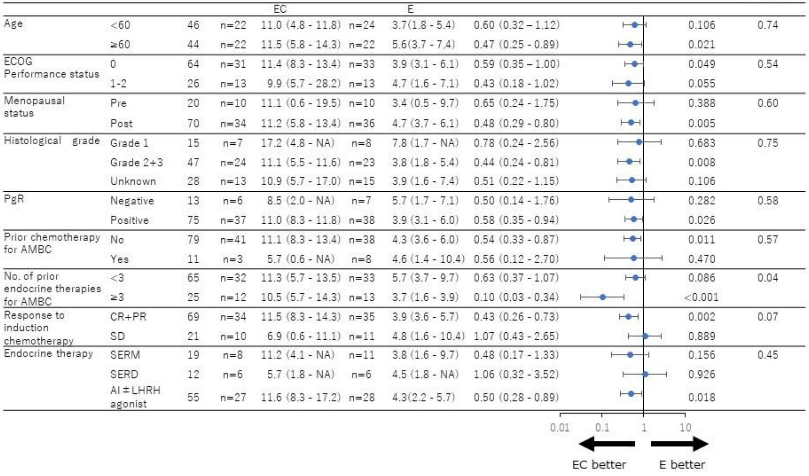

Figure S1. Forest plot for progression-free survival (PFS, months) in patients who received maintenance therapy with endocrine therapy alone (E) or endocrine therapy plus capecitabine (EC). AI, aromatase inhibitor; AMBC, advanced and metastatic breast cancer; CI, confidence interval; CR, complete response; ECOG, Eastern Cooperative Oncology Group; HR, hazard ratio; LHRH, luteinizing hormone–releasing hormone; PgR, progesterone receptor; PR, partial response; SD, stable disease; SERD, selective estrogen receptor down-regulator; SERM, selective estrogen receptor modulator.

		No. of patients		OS (95%CI)		OS (95%CI)	HR (95%CI)	P-value	Interaction test P-value
				EC		E			
Age	< 60	46	n=22	43.2 (25.7 - NA)	n=24	27.8 (20.7 - NA)	0.79 (0.36 - 1.71)	0.548	0.88
	≥ 60	44	n=22	64.0 (33.7 - NA)	n=22	47.2 (20.8 - NA)	0.81 (0.34 - 1.94)	0.641	
ECOG Performance status	0	64	n=31	43.8 (30.8 - NA)	n=33	47.2 (22.5 - NA)	0.98 (0.48 - 1.98)	0.949	0.34
	1-2	26	n=13	34.5 (13.7 - NA)	n=13	32.4 (12.6 - 36.0)	0.54 (0.18 - 1.62)	0.273	
Menopausal status	Pre	20	n=10	43.2 (19.4 - NA)	n=10	NA (6.1 - NA)	1.10 (0.24 - 4.91)	0.906	0.45
	Post	70	n=34	43.8 (33.7 - NA)	n=36	30.0 (22.5 - 57.8)	0.76 (0.41 - 1.43)	0.400	
Histological grade	Grade1	15	n=7	40.0 (25.4 - NA)	n=8	29.6 (7.2 - NA)	0.73 (0.19 - 2.73)	0.634	0.71
	Grade2+3	47	n=24	43.2 (30.8 - NA)	n=23	36.0 (23.4 - NA)	0.84 (0.36 - 1.96)	0.692	
	unknown	28	n=13	NA (20.5 - NA)	n=15	21.0 (14.2 - NA)	0.55 (0.18 - 1.65)	0.286	
PgR	Negative	13	n=6	37.0 (7.8 - NA)	n=7	NA (7.2 - NA)	1.62 (0.36 - 7.29)	0.527	0.29
	Positive	75	n=37	43.8 (33.7 - NA)	n=38	34.9 (23.4 - NA)	0.79 (0.42 - 1.49)	0.458	
Prior chemotherapy for AMBC	No	79	n=41	43.8 (34.5 - NA)	n=38	36.0 (22.5 - NA)	0.91 (0.48 - 1.73)	0.773	0.99
	Yes	11	n=3	25.7 (13.7 - NA)	n=8	25.6 (8.3 - NA)	0.93 (0.19 - 4.63)	0.927	
No. of prior endocrine therapies for AMBC	< 3	65	n=32	43.4 (28.0 - NA)	n=33	57.8 (22.5 - NA)	1.21 (0.61 - 2.40)	0.578	0.03
	≥ 3	25	n=12	65.5 (34.5 - NA)	n=13	23.4 (17.6 - 47.2)	0.19 (0.04 - 0.86)	0.031	
Response to induction chemotherapy	CR+PR	69	n=34	43.8 (33.7 - NA)	n=35	29.2 (21.0 - NA)	0.67 (0.34 - 1.31)	0.236	0.30
	SD	21	n=10	43.2 (7.8 - NA)	n=11	57.8 (8.0 - NA)	1.31 (0.42 - 4.12)	0.645	
Types of endocrine therapy	SERM	19	n=8	43.8 (19.4 - NA)	n=11	NA (17.6 - NA)	1.02 (0.23 - 4.60)	0.975	0.85
	SERD	12	n=6	16.3 (7.8 - NA)	n=6	25.5 (19.8 - NA)	1.39 (0.30 - 6.41)	0.673	
	AI ± LHRH agonist	55	n=27	46.2 (30.8 - NA)	n=28	34.9 (20.7 - NA)	0.73 (0.34 - 1.56)	0.416	

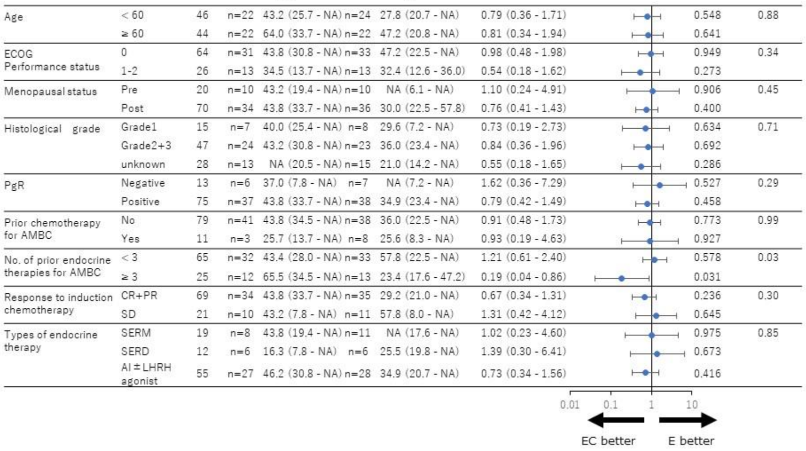

Figure S2. Forest plot for overall survival (OS, months) from the start of bevacizumab–paclitaxel induction therapy in patients who received maintenance therapy with endocrine therapy alone (group E) or endocrine therapy plus capecitabine (group EC). AI, aromatase inhibitor; AMBC, advanced and metastatic breast cancer; CI, confidence interval; CR, complete response; ECOG, Eastern Cooperative Oncology Group; HR, hazard ratio; LHRH, luteinizing hormone–releasing hormone; PgR, progesterone receptor; PR, partial response; SD, stable disease; SERD, selective es-trogen receptor down-regulator; SERM, selective estrogen receptor modulator.
